# Draft Genome Sequence of a Novel Picornavirus Isolated from Japanese Eel (*Anguilla japonica*)

**DOI:** 10.1128/MRA.00878-20

**Published:** 2020-10-15

**Authors:** Sachiko Terashima, Motoo Suzuki, Tomokazu Takano, Motoshige Yasuike, Tomomasa Matsuyama, Yuta Matsuura, Chihaya Nakayasu

**Affiliations:** a Pathology Division, Aquaculture Research Department, Fisheries Technology Institute, Japan Fisheries Research and Education Agency, Minami-Ise, Mie, Japan; b Hamanako Branch, Shizuoka Prefectural Research Institute of Fishery and Ocean, Hamamatsu, Shizuoka, Japan; c Bioinformatics and Biosciences Division, Fisheries Resources Institute, Japan Fisheries Research and Education Agency, Yokohama, Kanagawa, Japan; DOE Joint Genome Institute

## Abstract

We report the draft genome sequence of a novel member of the order *Picornavirales* that was obtained from the gills of farmed Japanese eel (Anguilla japonica). A putative polyprotein encoded by the genome was similar to that of other picornaviruses and shared 31% amino acid identity with that of eel picornavirus 1.

## Abstract (precis)

We report the draft genome sequence of a novel member of the order *Picornavirales* that was obtained from the gills of farmed Japanese eel (Anguilla japonica). A putative polyprotein encoded by the genome was similar to that of other picornaviruses and shared 31% amino acid identity with eel picornavirus 1.

## ANNOUNCEMENT

The Japanese eel (Anguilla japonica) is a widely cultured fish species in East Asia, and the cultivation of this species is dependent on seeds from nature. To reduce the pressure on natural stocks, methods such as broodstock domestication on a commercial scale have been studied extensively (1–3). Muroga reported the occurrence of vertical infection of seed by infected wild broodstock (4). Here, we describe a novel picorna-like virus that was isolated from eels reared from wild seed.

Eel picornavirus Aj/31945 was isolated from the gills of seven (numbers 1, 2, and 4 to 8) of eight Japanese eels farmed in Shizuoka, Japan, and collected in 2019. Gill tissues were preserved individually in RNAlater (Sigma-Aldrich), and total RNA was extracted from each of the samples using ISOGEN (Nippon Gene). Each RNA sample was purified using NucleoSpin RNA purification (TaKaRa Bio) with the TURBO DNase-free kit (Thermo Fisher Scientific) according to the manufacturers’ instructions. The sequencing libraries were prepared using the SMART-Seq stranded kit (TaKaRa Bio) and sequenced with 2 × 150-bp paired-end reads on an Illumina NovaSeq 6000 system. This study was approved by the Institutional Animal Care and Use Committee of the National Research Institute of Aquaculture (permission number IACUC-NRIA 20004).

The initial quality of each sequencing run was assessed using the quality control (QC) report tool of CLC Genomics Workbench v20.0.3 (CLC bio). Based on the initial quality reports, raw sequence reads were trimmed using the trim sequence tool of CLC Genomics Workbench v20.0.3 to remove the indexing adapters. After the trimmed reads were mapped to the draft genome sequence of the Japanese eel (GenBank accession numbers BEWY01000001 to BEWY01083292) using the mapping reads to reference tool of CLC Genomics Workbench v20.0.3, the unmapped reads from all eight samples were combined and assembled into contigs using the default *de novo* assembly strategy implemented in CLC Genomics Workbench v20.0.3. The contigs were analyzed with a BLASTX search against the nonredundant protein sequence database to determine their origins. For the contig identified as a picornavirus, coding sequences were predicted and compared to the nonredundant protein sequence database with BLASTp.

After trimming, the numbers of reads for the eel samples (numbers 1 to 8) were 3,893,934 (number 1), 2,921,006 (number 2), 798,534 (number 3), 2,772,532 (number 4), 703,022 (number 5), 576,022 (number 6), 529,572 (number 7), and 439,104 (number 8). *De novo* assembly generated 53,840 contigs, one of which was a 7,707-nucleotide contig with one contiguous open reading frame (ORF) that was similar to that of eel picornavirus 1 strain F15/05 (GenBank accession number NC_022332) (E value of <1E−300) (5), with a G+C content of 48%. This contig was named eel picornavirus Aj/31945. A total of 19,737 reads from the seven eel samples (numbers 1, 2, and 4 to 8) were mapped to the contig of eel picornavirus Aj/31945, providing an average coverage of 384×. The putative ORF encoded 2,194 amino acids. BLASTp analysis of the deduced amino acid sequence revealed that the greatest similarities for eel picornavirus Aj/31945 were 31.8% relative to a polyprotein of Beihai conger picornavirus (GenBank accession number AVM87437.1) (6) and 31.4% relative to a polyprotein of eel picornavirus 1 (GenBank accession number YP_008531322.1) (5).

### Data availability.

The draft genome sequence and raw reads reported here were deposited in DDBJ/ENA/GenBank under the accession numbers LC556994 and DRX232195, respectively.
